# *Xanthomonas oryzae *pv *oryzae *triggers immediate transcriptomic modulations in rice

**DOI:** 10.1186/1471-2164-13-49

**Published:** 2012-01-31

**Authors:** Rumdeep K Grewal, Sumanti Gupta, Sampa Das

**Affiliations:** 1 Division of Plant Biology, Bose Institute, Centenary Campus, P1/12 C.I.T. Scheme VII M, Kankurgachi, Kolkata-700054, India

## Abstract

**Background:**

*Xanthomonas oryzae *pv *oryzae *is a devastating pathogen of rice and has been extensively studied as a model pathogen of monocotyledons. Expressional studies in both the contenders have been undertaken in past to understand the molecular mechanism underlying the compatible and incompatible interactions in the pathosystem. Continuous update on database and gene annotations necessitates constant updating on the roles of the new entities as well as reinterpretation of regulations of the previous ones. Moreover the past endeavors have addressed the middle or late defense responses of the rice plant whereas in the present study an attempt has been made to investigate the early defense responses taking place immediately after inoculation.

**Results:**

Microarray was used to study the transcriptional modulations in eighteen days old rice seedling leaves of both susceptible and resistant genotypes one hour after inoculation. In resistant plants as compared to susceptible ones 274 genes were found to be differentially expressed. Annotations could be assigned to 112 up- and 73 down-regulated transcripts and gene interaction maps were generated for 86 transcripts. Expressional data and interaction maps were used to develop a hypothetical scheme of the molecular events taking place during early defense response. Network analysis with the differential transcripts showed up-regulation of major clusters of cell signaling proteins and transcription factors while growth and basal metabolic components were largely found to be down-regulated.

**Conclusions:**

This study provides an understanding of the early defense signaling in rice cells. Components of the calcium and lipid signaling as well as MAPK cascade were modulated, by signals from surface receptors and cytosolic R-proteins, to arouse jasmonic acid and ethylene signaling and suppress auxin signaling through various transcription factors. Abscisic acid modulation was also evident through the expression regulation of transcription factors involved with its functions. Moreover adjustments in expression levels of components of primary as well as secondary metabolism, protein trafficking and turnout were apparent, highlighting the complexity of defense response.

## Background

Plants interact with the environment in various ways and routinely face challenges from potential pathogens, but disease occurs only in limited cases as survival is a rule rather than an exception. Plants are sessile and unlike animals do not have mobile defender cells, instead they depend upon the innate immunity of each cell. Plants have preformed physical and chemical barriers and continuously produce antimicrobial compounds which are enough to deter most microbes, yet a pathogen may overcome these defenses and cause infection. When a potential pathogen gets over these barriers and is recognized by plant cells as an invader, a rapid and coordinated induction of defense response by resistant plant prevents microbe colonization and disease development, often termed as incompatible interaction. However if the plant is unable to recognize the pathogen or does not respond rapidly enough as in case of susceptible plants, disease spreads and is termed as compatible interaction. Recognition of the pathogen by plant triggers signal transduction cascades that leads to rapid defense mobilization [1,2]. R-gene products have long been implicated as the receptors which directly [3] or indirectly [4] recognize pathogens and initiate defense response. Conversely studies have shown that there are two branches of immune system, one uses transmembrane pattern recognition receptors (PRRs) that respond to pathogen associated molecular patterns (PAMPs) and the second, that acts largely inside the cell, uses NB-LRR type R-gene products [5]. Perception of an invader by host cell leads to activation of protein Kinases or/and inhibition of protein phosphatases triggering the Ca^2+ ^influx which in turn leads to active oxygen species (AOS) generation, MAPK activation, anion effluxes and plasma membrane depolarization [6]. The prime target of such signal transduction is the cell nucleus, where modulation of numerous genes takes place to face the invasion. The genes are coordinately activated in several waves [7]. The products of immediately activated genes or primary response genes subsequently activate the secondary response genes [8]. These subsequent transcriptional events reinforces and amplifies defense signals and results in production of antimicrobial metabolites, pathogenesis related proteins, enzymes of oxidative stress protection, stress related hormones, cell wall lignification and fortification and often hypersensitive response.

Rice (*Oryza sativa *L.) is the major nutritional source for above 60 percent of the global and 90 percent of the Asian population [9]. It globally provides 21 percent of human per capita energy and 15 percent of per capita protein http://www.knowledgebank.irri.org. But the crop yield obtained is greatly affected by various diseases of which blast, leaf blight and sheath blight are most devastating ones, resulting in a huge gap between the yield potential and the actual yield. Following blast, bacterial leaf blight of rice, caused by *Xanthomonas oryzae *pv oryzae (Xoo), is responsible for huge economic loss. Bacterial leaf blight is known to occur in all rice growing areas and is exceptionally severe in Asia. It is reported to have reduced annual production by as much as 60 percent in India and 50 percent in Japan http://www.knowledgebank.irri.org.It has also been extensively studied as a model disease of rice to understand the host-pathogen interactions, bacterial pathogenesis and defense responses in monocotyledonous plants [10]. The symptoms in adult plants appear as water-soaked yellowish stripes on leaf blades or starting at leaf tips which increase in length and width killing the infected leaves. Infected plants produce sterile and empty panicles and in severe cases the plant wilts and dies. At seedling stage the disease totally eradicates the plants of wide areas leading to epidemic. Enormous effort has been put to develop resistant cultivars carrying major R-genes of which 29 have been identified till date [10]. Availability of genome sequences for both rice and Xoo as well as continuing annotation projects has opened up the path for global expression studies of both contenders. Microarray technology has been excellently used to study constitutive and early defense responses in the concerned system. Previous studies have highlighted the complexity of the genetic networks involved in defense response. Ethylene and Jasmonic acid as well as MAPK pathways have been found to be important in case of rice-Xoo interaction.

The previous studies report transcriptomic events at four hours or later after inoculation [11,12] whereas considering the rapidity of plant defense response documented in other plant-pathogen systems [13] and short generation time of Xoo, it is expected that the bacterial pathogen induces host reprogramming even at prior time points. Moreover large accumulation of additional annotation data since the afore-mentioned studies is enough to justify a revisit to the problem. In the present study, to best of our knowledge, an effort has been made for the first time to dissect the rice-bacterial interaction system at one hour after inoculation (hai) in both resistant and susceptible hosts. This time point was selected after deliberate consideration of the facts that Xoo is known to reach early log phase in culture within one hour of growth [14] and the method of inoculation used i.e. the clipping method, deposits the pathogen directly in the infection court [15]. In parsley and bean cell suspension cultures, the phenylpropanoid biosynthetic gene transcription rate was found to be maximum at around one hour when challenged with avirulent pathogen [16] The present study delineates the early transcriptomic changes in response to pathogen attack in much detail and underlines the sophisticated regulatory mechanisms that are brought into play to combat the microbe invasion.

## Results and discussion

### Disease symptoms in IET8585 and IR24

In fifty five days old plants inoculated with Xanthomonas oryzae pv oryzae strain Bxo43, the symptoms first appear five days after inoculation (dai) as yellowish lesions around the site of inoculation in both the cultivars. In susceptible IR24 the whole leaf turned grayish yellow and dried up at 14 dai, lesions were visible on other leaves of the same plant as well. In resistant IET8585 at 14 dai the progression of lesion was limited to 16 +/- 2 cm from inoculation site whereas other leaves of the plant remained unaffected (Additional file 1). The symptoms in eighteen days old plants were also comparable with previously documented reports [12].

### Microarray experiment and validation

**Figure 1:**
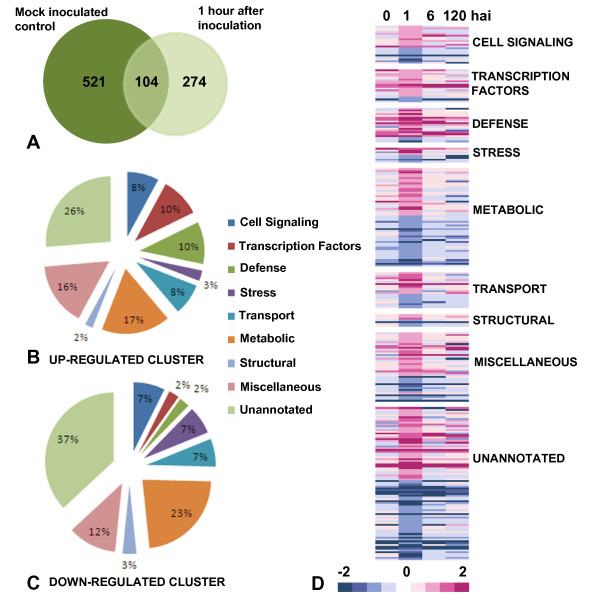
**The differentially expressed transcripts**. **A**. Vein diagram representing overlap of total transcripts between mock treated and pathogen treated samples. **B**. Functional categorization of up-regulated transcripts. **C**. Functional categorization of down-regulated transcripts. **D**. Heat map comparing the expression of transcripts at different time points i.e. 0,1,6,120 hours after inoculation. All data was generated using Agilent custom oligo Rice DNA chips using same experimental strategy and Loess normalization method. Magenta and blue represent up-regulation and down-regulation of transcripts respectively. The intensity of colors signifies the degree of fold changes.

Agilent Rice gene expression microarrays were used to examine differential transcript accumulation in resistant IET8585 and susceptible IR24 cultivars at 1 hai with Bxo43 or mock water treated control. The number of transcripts found to be differentially expressed in resistant plants compared to susceptible ones after pathogen inoculation were 378. Amongst them 104 were found to be differentially expressed after mock treatment as well and were not taken into consideration for further analysis (Figure 1A). A remaining subset of 274 transcripts were taken to be differentially expressed due to Bxo43 inoculation, of these 152 were found to be up-regulated and 122 down-regulated in IET8585 as compared to IR24. The microarray data has been submitted to ArrayExpress and is available as E-MEXP-3388.

Real-time qPCR was used to validate the microarray results. Fifteen genes were randomly selected from the differentially expressed genes. The differential expressions of selected genes were verified by quantitative PCR. Both the up- and down-regulated genes showed same trend of expression as obtained by microarray when further analyzed by qPCR (Additional file 2).

### Identification and annotation of differentially expressed transcripts

The transcripts were annotated with the help of Rice Annotation Project Database http://rapdb.dna.affrc.go.jp. Annotations could be assigned to 112 up- and 73 down-regulated transcripts. On the basis of GO functional categories transcripts were assorted into several groups. The up-regulated transcripts were grouped into cell signaling (8%), transcriptional factors (10%), defense related (10%), general stress related (3%), transport (8%), metabolic (17%), structural (2%) and miscellaneous (16%) (Figure 1B, Additional file 3). Similarly down-regulated transcripts consisted of cell signaling (7%), transcription factors (2%), defense related (2%), stress related (7%), transport (7%), metabolic (23%), structural (3%) and miscellaneous (12%) (Figure 1C, Additional file 4). Interactions for 55 up-regulated (Additional file 5) and 31 down-regulated (Additional file 6) transcripts were mapped.

### Expression of modulated transcripts at different time points

The present 1 hai data set was compared to previously reported expressional studies done at 6 and 120 hai as well as in untreated plants-GEO Data set GSE6244 [12]. Amongst the 274 transcripts obtained from present study 233 showed similar trend in 6 or 120 hai data sets, further 172 showed greatest differential expression at 1 hai, 17 were constitutively up-regulated and 22 were constitutively down-regulated in all data sets (Figure 1D, Additional file 7). Constitutively up-regulated transcripts include WRKY69, DREB1B, chitinase precursor, Hin1 and NB-ARC domain containing proteins (Table 1) while constitutively down-regulated transcripts include Phospholipase A_2 _(Table 2). Transcripts which were uniquely up-regulated at 1 hai include calmodulin like protein, Nod 19 family protein and Protein disulphide isomerase. The comparison of expression data at different time points revealed that a plant maintains a continuous state of alertness as soon as it perceives a pathogen attack and coordinately modulates the expression of genes for the purpose. While the differential expression of some genes i.e. chitinase, WRKY factors, DREB1B, Phospholipase, Nod family proteins may provide an advantage against pathogen, over-expression of NB-ARC domain containing or calmodulin-like protein may help in rapid perception and signal transmission.

**Table 1:** A list of up-regulated transcripts in resistant IET8585 as compared to susceptible IR24 one hour after inoculation with Bxo43

Functional Category	Gene Name	Fold change	q-value	Gene Description
Cell Signaling	AK068504	2.497697	0.036	Similar to Receptor-like protein kinase.
	AK111766	2.126773	0.023	Similar to LysM domain-containing receptor-like kinase 3.
	AK071585	3.051916	0.021	Serine/threonine protein kinase domain containing protein.
	AK068725	2.730174	0.029	Mitogen activated protein kinase kinase kinase 3 domain containing protein.
	AK100561	2.068673	0.036	Similar to Protein phosphatase 2C gamma isoform (EC 3.1.3.16) (PP2C-gamma) (Protein phosphatase magnesium-dependent 1 gamma) (Protein phosphatase 1C) (Fibroblast growth factor inducible protein 13) (FIN13).
	AK070889	2.007305	0.017	Similar to Calmodulin-like protein.
	AK110372	2.442006	0.015	Calmodulin binding protein-like family protein (Os12g0556500).
	AK111852	2.789168	0.017	Similar to EF-hand Ca2+-binding protein CCD1.(Os06g0683400)
Transcription Factor	AK105817	2.030093	0.017	Similar to MYB transcription factor R2R3 type.
	AK099506	2.210962	0.022	Similar to Transcription factor MYC7E (Fragment).
	AK068232	3.077906	0.174	AUX/IAA protein family protein.
	AK073848	2.138125	0.017	Similar to NAC domain protein.
	AK062422	7.448169	0.017	Dehydration-responsive element-binding protein 1B.
	AK111606	3.783378	0.044	WRKY transcription factor 69.
	AK073812	2.587758	0.017	APETALA2 (AP2) gene 59., Abiotic stress., AP2/ERF family protein.
Defense related	AK069420	14.04906	0.020	NB-ARC domain containing protein.
	AK100906	4.244798	0.019	Similar to Diacylglycerol kinase.
	AK099489	2.399005	0.048	Similar to Glutathione S-transferase GST 23 (EC 2.5.1.18) (Fragment).
	AK063796	3.123453	0.173	Similar to Glutathione S-transferase GST 8 (EC 2.5.1.18).
	AK071599	2.625772	0.225	Similar to Cytochrome P450 71A1 (EC 1.14.-.-) (CYPLXXIA1) (ARP-2).
	AK064764	2.834801	0.047	Cytochrome P450 family protein.
	AK107349	3.065706	0.047	Similar to Cytochrome P450 monooxygenase CYP72A5 (Fragment).
	AK104472	6.697833	0.017	Similar to flavanoid 3-monoxygenase
	AK099355	4.241933	0.017	Similar to Chitinase (EC 3.2.1.14) (Fragment).
	AK100973	8.113719	0.019	Acidic class III chitinase OsChib3a precursor (Chitinase) (EC 3.2.1.14).
	AK068115	6.352779	0.017	Harpin-induced 1 domain containing protein.
	AK108457	3.262299	0.183	Harpin-induced 1 domain containing protein.
	AK066825	10.15681	0.017	Similar to Lipoxygenase, chloroplast precursor (EC 1.13.11.12).
Stress	AK061337	10.75691	0.100	Similar to Flavanone 3-hydroxylase-like protein
	AK069823	2.294991	0.050	Similar to DnaJ homolog subfamily B member 1 (Heat shock 40 kDa protein 1) (Heat shock protein 40) (HSP40) (DnaJ protein homolog 1) (HDJ-1).
	AK068268	3.10078	0.026	Similar to Protein disulfide isomerase (Fragment).
Transport	AK066194	4.009016	0.017	Similar to High-affinity potassium transporter.
	AK100411	3.405603	0.050	Ammonium transporter.
	AK064899	3.501921	0.050	Similar to Peptide transporter PTR2-B (Histidine transporting protein).
	AK099079	3.654256	0.019	Similar to Monosaccharide transporter 3.
	AK073216	4.20715	0.017	Similar to Sorbitol transporter.
	AK069583	2.063748	0.017	Similar to PDI-like protein.
Metabolic	AK061884	3.698292	0.017	Similar to Nucellin-like aspartic protease (Fragment).
	AK066720	2.718419	0.017	Lipase, class 3 family protein.
	AK070038	4.264953	0.018	Similar to RING-H2 finger protein ATL1R (RING-H2 finger protein ATL8).
	AK071972	3.545375	0.017	Similar to Short-chain dehydrogenase Tic32.
	AK100909	3.13932	0.017	Similar to 1-deoxy-D-xylulose 5-phosphate synthase 2 precursor.
	AK059608	2.516794	0.209	Similar to Nuclease I.
	AK061438	3.693648	0.178	Ribonuclease T2 family protein.
	AK071652	2.787926	0.039	Beta 5 subunit of 20S proteasome.
Structural	AK103678	2.167948	0.017	Ribosomal protein S8e domain containing protein.
	AK068395	2.523953	0.017	Actin/actin-like family protein.
	AK107269	3.393664	0.035	Ribosomal protein S14, conserved site domain containing protein.
Miscellaneous	AK107102	3.477243	0.155	Similar to F-box/LRR-repeat MAX2 homolog.

**Table 2:** A list of down-regulated transcripts in resistant IET8585 as compared to susceptible IR24 one hour after inoculation with Bxo43

Functional Category	Gene Name	Fold change	q-value	Gene Description
Cell Signaling	AK100131	5.114667	0.027	Phox-associated domain domain containing protein.(Os05g0583500)
	AK100302	2.005169	0.024	EF hand domain containing protein.
	AK060253	2.242858	0.204	Leucine-rich repeat 2 containing protein.
	AK060392	3.321018	0.018	Remorin, C-terminal region domain containing protein.
	AK109657	2.541251	0.018	WD40 repeat-like domain containing protein.
Transcription Factor	AK099793	2.577764	0.040	Similar to Auxin response factor 5.
	AK066518	2.074774	0.174	AUX/IAA protein family protein.
	AK059464	3.665959	0.152	Transcriptional factor B3 family protein.
Transport	AK066544	2.491852	0.152	Similar to Potassium transporter 22.
	AK069303	2.290848	0.017	Similar to K+ channel protein.
	AK061365	2.073459	0.024	Similar to BS14b.
Metabolic	AK071503	2.522627	0.214	Similar to ASF/SF2-like pre-mRNA splicing factor SRP31'''.
	AK105828	3.215238	0.062	Phospholipase A2 family protein.
	AK062946	3.382547	0.017	Nucleotide excision repair, TFIIH, subunit TTDA domain containing protein.
	AK103277	3.693126	0.114	Similar to RNA-binding protein 8A (Tsunagi protein).
	AK111609	2.187418	0.024	Methyltransferase type 12 domain containing protein.
	AK069606	2.597207	0.017	Similar to adenine phosphoribosyltransferase 2.
	AK060566	2.141265	0.172	Similar to Acyl carrier protein (ACP).
Structural	AK060420	2.015343	0.017	Similar to 30S ribosomal protein S31, chloroplast (Fragment).

### Cell signaling

It was interesting to note that the largest up-regulated cluster consisted of 15 transcription factors and 12 cell signaling related proteins, transcripts which or the products of which may act as mediators to usher defense response. In the corresponding down-regulated cluster 3 transcription factors and 9 signaling related proteins were found. Two receptor-like kinases (AK068504, AK111766) were found to be up-regulated (Table 1), one of them bearing a LysM domain (AK111766). The LysM domains are found in a variety of peptidoglycan or chitin binding proteins and have been implicated in perception of rhizobial lipichitooligosaccaride signals [17] and further elicitation of signals via its intercellular kinase domain [18]. A transcript for NB-ARC domain containing protein (AK069420) was found to be highly up-regulated (Table 1) as well. NB-ARC is an ancient highly conserved domain of a class of plant resistance proteins [19]. It has a functional ATPase domain and its nucleotide binding site is proposed to regulate activity of the R-protein in pathogen recognition.

**Figure 2:**
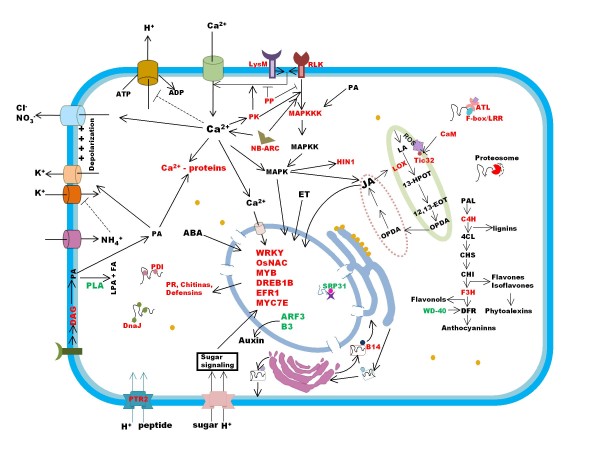
**Schematic representation of molecular change in rice cell during early defense response**. The up-regulated proteins are labeled in red and down-regulated ones in green.

Serine/theonine protein kinases (STK) have long been implicated to play a role in signaling processes concerned with self verses non-self recognition and disease resistance [20]. A up-regulated STK was found to be similar to MAPKKK17 (AK071585) (Additional file 5), which is known to be induced by pathogens [21]. Another MAPK cascade initiating protein MAPKKK3 (AK068725) [22] as well as Protein Phosphatase 2C (AK100561) or PP2C (Additional file 5) a regulator of MAPK pathway, known to be activated in stress [23], was found to be up-regulated in the present case study, suggesting that there may be a remarkable fine tuning of MAPK cascade at such early a time point. Calcium ion is the most important signal entity in cell, its importance is reflected in present work by differential regulation of transcripts of several associated proteins, up-regulation of a calmodulin-like protein (AK070889), Os12g0556500 (AK110372) a calmodulin-binding protein-like protein and Os06g0683400 (AK111852) an EF-hand domain containing protein, similar to CCD1 (Table 1). Studies in wheat cultured cells have revealed that *ccd-1 *mRNA are strongly responsive to elicitors of snow mold and gene product CCD1 plays a role in elicitor provoked Ca^2+ ^mediated signal transduction [24]. Some Ca^2+ ^binding proteins Os05g0583500 (AK100131) a calcium binding protein and an EF-hand domain containing protein (AK100302) were also found to be down-regulated indicating tight regulation of signaling pathways. Besides Ca^2+ ^flux transporters for several other ions across membrane were also stimulated. These ion fluxes affect membrane potential which in turn affect uptake by other channels and activation of defense response [25]. A net K^+ ^eflux in elicitor treated cells is a common early response [26]. In present data set a transcript similar to high affinity K^+ ^uptake pump HAK5 (AK066544) (Additional file 6) [27] and K^+ ^channel protein (AK069303) (Table 2) were found to be down-regulated whereas a low to high affinity K^+ ^pump KUP3 (AK066194) [28] is up-regulated implying a regulation of K^+ ^uptake. An additional complexity is introduced by the fact that K^+ ^uptake is sensitive to ammonium transport and a transcript for AMT2-like protein (AK100411) an ammonium transporter has also been found to be up-regulated [29,30]. Proton influx is another important defense response and three H^+ ^transporters were found to be up-regulated in present study (Additional file 5), one of them was a proton-peptide symporter PTR2 (AK064899) and other two were sugar-proton symporters STP1 (AK099079) and PLT5 (AK073216) like proteins. The sugar-H^+ ^symporters probably serve dual purpose, they relocate H^+ ^and sugars into infected cells, which behaves as a sink and draws carbon resources for energy consumption to put up defense on one hand [31,32] while on the other hand modulate sugar signals (Figure 2) which are known to influence SAR pathway [33,34]. Monosaccharide-H^+ ^symportes in *Arabidopsis *are rapidly induced by pathogenic elicitors [31].

Lipid signaling has emerged as an important component of stress signaling and Phospatidic acid (PA) is a key lipid second messenger. PA is directly formed via activation of Phospholipase D (PLD) and indirectly by phosphorylation of Diacylglycerol (DAG) by DAG kinase (AK100906) or DGK (Additional file 5) [35]. The DGK pathway has been known to be activated in defense response against pathogens within few hours of infection [36], a transcript similar to PA generating DAG kinase is upregulated in the present data set as well whereas a phospholipase A2 family protein (AK105828) was down-regulated (Additional file 6). PA can be deacylated by phospholipase A2 (PLA2) to produce LPA and free fatty acids (Figure 2), which are signaling compounds in plant responses to auxin [37]. Further a transcription factor Auxin responsive factor 5 or ARF5 (AK099793) that binds to AUX/IAA family proteins (Additional file 6), a critical component in auxin signaling pathway, as well as an AUX/IAA family protein (AK066518) were found to be down-regulated [38], incidentally another AUX/IAA family protein (AK068232) was found to be up-regulated (Additional file 5). This two-way modulation of AUX/IAA family proteins is in agreement with previous findings. AUX/IAA genes were discovered based on their induction by auxin and some can promote auxin response, however many AUX/IAA proteins actually inhibit auxin response [39]

The down-regulated cell signaling associated transcripts include two interesting genes a Leucine-rich repeat 2 containing protein (AK060253) and a Remorin domain containing protein (AK060392) (Table 2). LRR repeats occur in various proteins to provide structural framework for protein-protein interaction and when associated to kinase domain are known to function as receptors. Remorins are unique to plants and are ubiquitously expressed. Differential expression of remorins have been well documented in *Arabidopsis*, interestingly most of the remorins were found to be down-regulated in case of bacterial-plant incompatible interactions [40].

### Transcription factors

The host cell gears up its arsenal on receiving signals of pathogen attack through elaborate signal transduction network by modulating expression of several transcription factors. These act as hubs that further modulate defense, hormone and growth related genes. The up-regulated cluster consists of several transcription factors some of them are R2R3 type MYB transcription factor (AK105817) related to stress response [41], MYC7E (AK099506) related to ABA response, OsNAC protein (AK073848), DREB1B(AK062422), Ethylene responsive factor (ERF1) family protein (AK073812), WRKY69 (AK111606) (Table 1). WRKY69 is a member of the well documented WRKY family related to regulation of many cellular processes including defense and is known in turn to be modulated by MAPK cascade [42]. OsNAC is induced by abiotic and biotic stress as well as ABA, Jasmonic acid and hydrogen peroxide [43,44] and plays a role in activation of defensin [45]. DREB1B responds to variety of stress thus inducing expression of PR genes [46]. ERF1 is induced by both ethylene and JA [47,48]. It integrates JA and ethylene signaling [49] and both signaling pathways are required simultaneously [50] for ERF1 induction. The tight co-regulation of ERF1 by both stress hormones is quite understandable considering the fact that of all the total number of defense related genes induced by ethylene and JA about 80% are ERF1 mediated. There are also reports of ERF1 being under MAPK cascade control mediated by EIN2 and EIN3 [51]. The most important genes up-regulated by ERF1 are basic chitinase, defensins and glutathione synthases (AK099489, AK063796) which have been found to be up-regulated in present study as well (Table 1). Ethylene and JA play a pivotal role in plant defense, plants with impaired ethylene signal transmission exhibit susceptibility to necrotrophic bacterium [52]. Ethylene induces cell-wall strengthening, xylem occlusion response, phenylpropanoid derived phytoalexin production, PR-genes including Beta-glucanase and chitinase [53] both of which have been found to be up-regulated in the present study. While Ethylene and JA have synergistic functions ABA is antagonistic to both, it interferes at different levels with ethylene and JA signaling [54]. The transcription factors found to be down-regulated were Auxin response factor 5, AUX/IAA and B3 transcription factor (Additional file 6). All three are growth related and are expressed in all organs, Auxin response factor 5 is required for normal growth [38] and B3 belongs to a family of plant transcription factors with various roles in plant development.

### Metabolism

Secondary metabolites play an important role in plant defense [55]. The present study also documents similar findings; there was up-regulation of seven genes related to flavanoid biosynthesis (Table 1), three cytochrome P450 monoxygenases (AK071599, AK064764, AK107349), a flavanoid 3-monoxygenase (AK104472), A flavanone 3-hydroxylase-like protein (AK061337) [56] and a Glutathione S-transferase-GST 23 (AK099489) (Figure 2). GST proteins are known to act as escort proteins in flavanoid transport. WD-40 repeat like domain containing protein (AK109657) was down regulated (Figure 2). WD-40 repeat containing protein have been found to be necessary for anthocyanin biosynthesis at the DFR (Dihydroflavonol reductase) step in *Arabidopsis *leaves but does not seem to affect upstream genes involved in flavanoid bio-synthesis. It appears that flavanoid biosynthesis pathway is modulated to produce excess flavanoides rather than anthocyanins. The flavanoides may produce lignins to strengthen cell wall or phytoalexins the classical anti-microbial plant compound [57]. Transcripts for several well studied pathogen induced genes were found to be up-regulated these include: a chitinase (AK099355), a chitinase precursor Oschib (AK100973), an aspartic protease Os11g0183900 (AK061884) as well as two Harpin-induced 1 (Hin1) domain containing proteins (AK068115, AK108457) (Table 1). Chitinases are induced by environmental stress and considered to play a role in active or passive defense [58,59]. Aspartic protease is a major family of protease enzymes, expression of an aspartic protease was found to be up-regulated in case of incompatible interaction between potato and the fungus *Phytophthora infestans *[60]. Hin1 is induced by bacterial effector, harpin through MAPK activity [61].

Genes related to lipid metabolism were also found to differentially regulated, a lipase (AK066720) (Additional file 5) was up-regulated whereas a gene related to lipid biosynthesis, an Acyl carrier protein (AK060566) was down-regulated (Table 2). The cell lipid metabolism perhaps is so diverted to provide ingredients and energy for mounting defense respose. A lipoxygenases (AK066825), chloroplast precursor of lipoxygenase LOX2 (Additional file 5), was also found to be significantly up-regulated. Lipoxygenases are key enzymes of lipid metabolism and JA biosynthesis [62]. LOX2 is required for wound induced JA accumulation and is involved in early defense response to pathogens [63]. The expression of LOX2 in turn is enhanced by JA through a positive feedback loop. The up-regulation of JA producing enzyme indicates the important role played by JA signaling in the *Xanthomonas*-rice incompatible interaction. Along with chloroplastid lipoxygenase other plastidial proteins including Tic32 (AK071972) and a protein similar to 1-deoxy-D-xylulose 5-phosphate synthase 2 precursor or DXPS (AK100909) were up-regulated (Additional file 5) as well. Tic32 is a NADPH-dependent dehydrogenase and its dehydrogenase activity is affected by Calmodulin. It is associated with Tic translocon on the stomatal side of the plastidial inner envelope. It may serve as a switch to differentially integrate redox signals from inside of chloroplast with calcium signals outside and influence the activity and/or specificity of Tic translocon [64,65].

Defense involves induction as well as repression of several proteins. In order to meet the demand the cell modulates several components of transcriptional, translational and post-translational modification machinery. Present work documents up-regulation of Nuclease1(AK059608), a Ribosomal T2 family protein (AK061438), Ribosomal protein S8e domain containing protein (AK103678), Ribosomal protein S14 domain containing protein (AK107269) and a protein related to ARP6 (AK068395) (Table 1). ARP6 is a component of chromatin modifying complex implicated in maintaining state of gene activation [66]. While several others were down regulated (Table 2), these included TFIIH domain containing protein (AK062946), methyltransferase type 12 domain containing protein (AK111609), RNA-binding protein 8A-like protein (AK103277), pre-mRNA slicing factor SRP31 (AK071503), ribosomal protein S31 (AK060420) and an adenine salvage related protein APRT2 (AK069606). Transcripts for F-box/LRR-repeat MAX2 homolog (AK107102) and RING-H2 finger protein ATL8 (AK070038) were found to be up-regulated. F-box/LRR-repeat proteins function as substrate recruiting subunit of SCF-type Ubiquitin E3 ligases [67]. ATL is a multigenic family of putative RING-type E3 ubiquitin ligases [68], the specificity determinants that mediate the transfer of ubiquitin to the ε-amino group of target proteins resulting in mono-ubiquitination, additional ubiquitin moieties are transferred to the target protein by E4, a multiubiquitin chain assembly factor [69]. While multi-ubiquitination generally tag proteins for degradation, mono-ubiquitination of a target results in non-proteolytic events such as changes in protein activity, histone modification, localization or protein-protein interactions [70]. Incidentally beta 5 subunit of 20S proteosome (AK071652), the core complex of the 26S proteasome, was also found to be up-regulated implying modulation of ubiquitin mediated protein degradation.

Protein transport apparently has also been affected, the relocation of proteins to new sites for defense was evident by the down-regulation of B14 protein (AK061365) (Table 2) involved in peptide transport from ER to golgi and up-regulation of a DnaJ like protein (AK069823), two peptide disulphide isomerases (PDI) like proteins (AK068268, AK069583) and a peptide-proton symporter PTR2 (Table 1). DnaJ and PDI are molecular chaperones and rapid induction of PDI in wheat after fungal inoculation during early response has been previously documented [71].

## Conclusion

Although massive efforts have been put in past to annotate and characterize gene functions, it was difficult to assign role to many differentially expressed transcripts due to lack of information. Moreover numerous transcripts have been annotated as proteins containing domains having diverse functional roles but their specific roles remain elusive. Nevertheless through analysis of present dataset and annotations of transcripts differentially expressed at 1 hai it was found that as the plant faces the pathogenic challenge it suspends its growth till it can spare the resources, thus there was up-regulation of defense related genes and loss of growth related ones. In resistant plant host cell recognizes the pathogen through plasma membrane or/and cytoplasm located receptors (Figure 2) and initiates diverse signaling pathways including MAPK cascade, Ca^2+ ^signaling, ionic fluxes, lipid and sugar signaling. These signals are ultimately transduced to nucleus resulting in up-regulation of several defense and stress related transcription factors and down-regulation of growth and development related ones. The transcription factors in turn modulate the expression of down-stream genes resulting in metabolic modulations. Thus ethylene and jasmonic acid responses are activated while auxin signaling is repressed (Figure 2). Protein turn-out and trafficking are adjusted to churn out a proteome suited to serve the defensive needs. Primary metabolites are channeled to provide energy and raw materials for defense and secondary metabolism is diverted to produce pathogen deterrents (Figure 2). Amongst the modulated transcripts the receptor like proteins i.e. LysM and NB-ARC domain containing proteins are attractive candidates as *R*-genes and hence also for transgenic modifications for resistance development. A Nod 19 family protein was another interesting transcript found to be up-regulated. This family of proteins has been implicated in nodule development but their role in pathogenesis remains elusive, in depth studies may reveal the specific advantage that this protein may provide to host cells in host-pathogen interaction. The gene modulations undertaken by plant cells at such early a stage highlight the ability of plant cells to rapidly mount a complex defense response. The resistant plant unleashes a diverse arsenal in a highly coordinated manner, no wonder that the susceptible plant which lags behind falls to disease.

## Methods

### Plant and bacterial culture

Seeds of bacterial blight resistant cultivar IET8585 [72] were obtained from Central Rice Research Institute Orissa, India and those of susceptible IR24 from Chinsurah Rice Research Station, West Bengal, India. Both varieties were grown on synthetic soil (Kaltech Energies, Karnataka, India) in a green house under 16 hrs light/8 hrs dark photoperiod at 28+/-2°C temperature.

The *Xanthomonas oryzae *pv *oryzae *culture Bxo43 [73] was obtained from Centre for Cellular and Molecular Biology, Andhra Pradesh, India.

### Inoculation

Eighteen and fifty-five days old plants were inoculated with Bxo43 by clipping method [74]. Sterile water treated plants served as mock control. Leaf samples from eighteen days old seedlings were collected one hour after inoculation (hai), flash frozen in liquid nitrogen and subjected to molecular analyses. Disease progression was studied in both adult plants and seedlings.

### RNA extraction and microarray hybridization

RNA from leaves of eighteen days old seedlings of both inoculated and mock-inoculated samples was extracted using tri-reagent (Sigma-Aldrich, St.Louis, MO, U.S.A.) and purified by Qiagen RNeasy Maxi Kit (Qiagen, Valencia, CA, U.S.A.) following manufacturers' instructions. The quality and purity of RNA was analyzed using spectrophotometer (Nano-Drop, Wilmington, DE, U.S.A.) and Agilent 2100 Bioanalyzer (Agilent Tecnologies, Santa Clara, CA, U.S.A.). Total RNA (200ng) was labeled with Cy5 or Cy3 using an Agilent Quick Amp Kit (Agilent Technologies).The amplified products were purified using Qiagen RNeasy Mini Kit (Qiagen), the recommended amount, 825 ng of each of the labeled products were used for array hybridization. Labeled targets of resistant and susceptible genotypes similarly treated (inoculated or mock inoculated) were hybridized to the same Agilent 44K custom oligo DNA microarray G2519F (Agilent). Dye-swap procedure was followed for two independent biological replicates (Additional file 8). Hybridization and wash processes were performed according to the instructions of the manufacturer. Microarrays were scanned using an Agilent Microarray Scanner (G256CA) at recommended settings (Additional file 9).

### Data analysis

Data from each of the four arrays was extracted using Agilent Feature Extraction 10.5.1.1 software following protocol recommended by the manufacturer. Raw data was exported to Genespring GX11 (Agilent Technologies). Signals were background corrected and baseline transformed to the median of all spots. The data was log2 transformed and normalized to 75^th ^percentile using Loess normalization. The log2 ratios were averaged for replicate spots. Saturated spots and oligonucleotides with more than fifty percent replicate spots flagged as absent were excluded from analysis. Differentially expressed genes were identified using Students unpaired t-test with a corrected p-value of < = 0.05 and fold change of two or above. Gene interaction pathways were generated with the help of the software Pathway Studio 7.1 (Ariadne Genomics,Rockville, MD, U.S.A.).

### Real-time qRT-PCR

RNA from independent biological replicate was used to synthesize cDNA employing Fermentas Revert Aid H minus first strand kit (Fermentas Life Sciences, Glen Burnie, MD, U.S.A.). Fifteen genes were randomly selected from among those that showed a significant up- or down-regulation in response to treatments. Specific primers (Additional file 10) were designed from the selected genes employing Primer3 software [75] and by comparison and alignment with available rice gene sequences from NCBI and Rice Annotation Project Database (RAP-DB). Actin [76] and Ubiquitin-conjugating enzyme E2 [77] were used as internal controls. PCRs were carried out in Bio-rad iQ5 Multicolor Real-Time PCR Detection System (Bio-rad Laboratories, Hercules, CA, U.S.A.) using iQ Syber Green Supermix (Bio-rad Laboratories) (Additional file 9). Quantification was based on cycle threshold (Ct value) and PCR efficiency determined by iQ5 Optical System Software 2.0 (Bio-rad Laboratories). The expression of each gene was normalized with internal controls and relative fold change was calculated using 2^-ΔΔCt ^method [78].

## List of abbreviations

ABA: Absicisic Acid; AMT: Ammonium transporter; APR: Actin related protein; ATL: Alkyltransferase-like protein; AUX/IAA: Auxin response/Indole-3-acetic acid induced proteins; CCD1: Coiled coil DIX domain; DREB1B: Drought responsive element binding protein 1B; HAK5: High affinity K^+ ^transporter 5; Hin1: Harpin induced protein1; KUP3: K^+ ^uptake transporter 3; LRR: Leucine rich repeats; LysM: Lysin motif; MAPK: Mitogen activated protein kinases; MAPKKK: MAPK kinase kinase; MAX2: More axillary branches 2 protein; NAC: NAM, ATAF1, 2, and CUC2 transcription factors family; NB-ARC: Nucleotide binding adaptor shared by APRF-1, R proteins and CED-4; PR-genes: Pathogenesis related genes; PTR2: Peptide transpoter 2; RING: Really interesting new gene; SCF: Skp, Cullin, F-box containing protein; SRP31: Serine/argentine-rich protein 31; STP1: Sugar transporter 1; Tic32: Translocon of the inner chloroplast membrane 32.

## Authors' contributions

RKG, SG and SD designed the experiments and drafted the manuscript. RKG performed the experiments. RKG and SD analyzed the data. RKG, SG and SD revised the manuscript. All authors have read and approved the final manuscript.

## Supplementary Material

Additional file 1 ***Xanthomonas oryzae *pv *oryzae *induced disease symptoms in adult rice plants**. **A**. Susceptible IR24. **B**. Resistant IET8585. Click here for file

Additional file 2 **Quantitative RT-PCR validation of microarray data**. A powerpoint file containing comparison of pathogen induced differential fold inductions as obtained from microarray and real-time PCR. Click here for file

Additional file 3 **Complete list of transcripts found to be up-regulated in resistant plant as compared to susceptible one**. Excel file containing the gene name, fold change and gene description of the transcripts. Click here for file

Additional file 4 **Complete list of transcripts found to be down-regulated in resistant plant as compared to susceptible one**. Excel file containing the gene name, fold change and gene description of the transcripts. Click here for file

Additional file 5 **Interaction network of up-regulated transcripts**. A powerpoint file containing interaction map generated by Pathway Studio (version 7.1). The transcripts up-regulated in present study are highlighted in yellow. Click here for file

Additional file 6 **Interaction network of down-regulated transcripts**. A powerpoint file containing interaction map generated by Pathway Studio (version 7.1). The transcripts down-regulated in present study are highlighted in yellow. Click here for file

Additional file 7 **K means clusters comparing the expressional level of transcripts**. A powerpoint file containing K means clusters comparing the expression level of transcripts having beyond +/-2 fold change and p < = 0.05 at 0,1,6 and 120 hours after inoculation. The green and orange represent down-regulation and up-regulation respectively. The intensity of color signifies the degree of fold change. Click here for file

Additional file 8 **Microarray experiment design**. Excel file containing the details of the samples hybridized to each array. Click here for file

Additional file 9 **The representative data of microarray hybridization and qRT-PCR**. A powerpoint file containing **A**. Part of image of microarray hybridization. **B**. Scatter plot of log intensities across an array. **C**. Real-time PCR curve. Click here for file

Additional file 10 **List of primer for the qRT-PCR**. Excel file containing all primer sequences used for the quantitative RT-PCR. Click here for file
