# Supplementary material for: Xanthomonas oryzae pv oryzae triggers immediate transcriptomic modulations in rice
Source: BMC Genomics. 2012 Jan 31;13:49. doi: 10.1186/1471-2164-13-49 (PMC3298507; doi:10.1186/1471-2164-13-49)
Supplement: Additional file 1 — Xanthomonas oryzae pv oryzae induced disease symptoms in adult rice plants. A. Susceptible IR24. B. Resistant IET8585. [file 1471-2164-13-49-S1.PPT]

## Slide 1
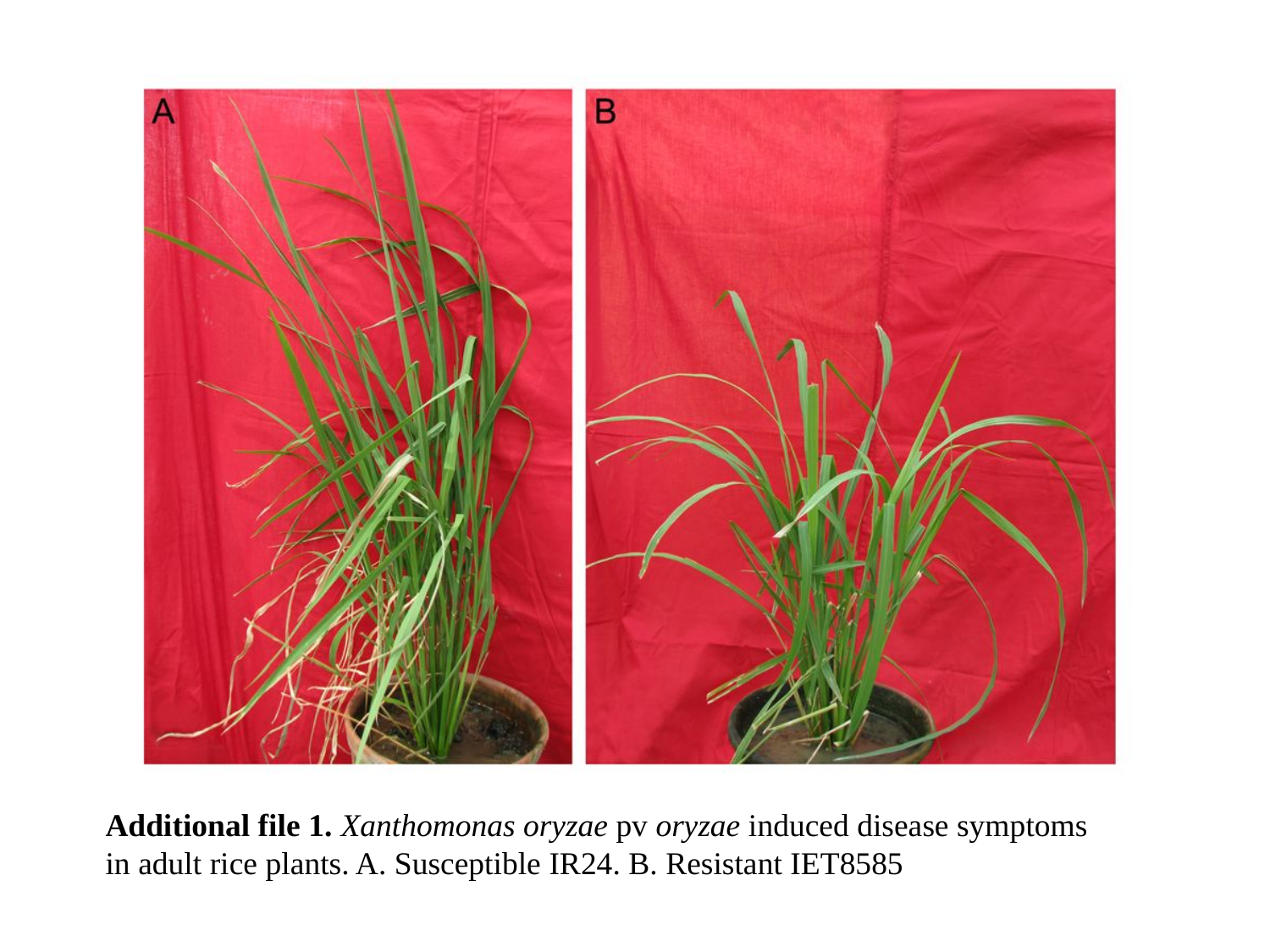

Additional file 1. Xanthomonas oryzae pv oryzae induced disease symptoms in adult rice plants. A. Susceptible IR24. B. Resistant IET8585
