# Supplementary material for: Xanthomonas oryzae pv oryzae triggers immediate transcriptomic modulations in rice
Source: BMC Genomics. 2012 Jan 31;13:49. doi: 10.1186/1471-2164-13-49 (PMC3298507; doi:10.1186/1471-2164-13-49)
Supplement: Additional file 2 — Quantitative RT-PCR validation of microarray data. A powerpoint file containing comparison of pathogen induced differential fold inductions as obtained from microarray and real-time PCR. [file 1471-2164-13-49-S2.PPT]

## Slide 1
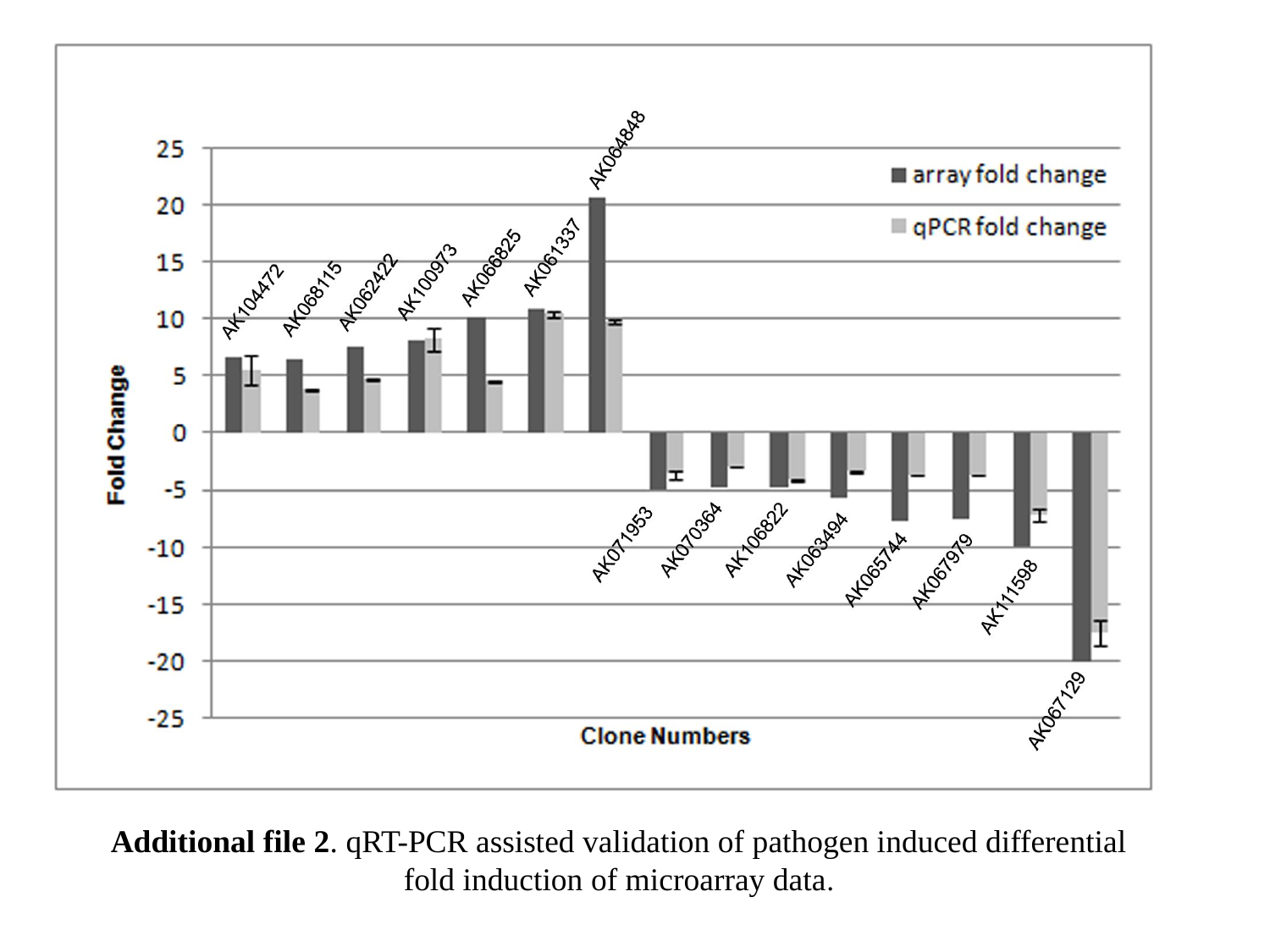

# Additional file 2. qRT-PCR assisted validation of pathogen induced differential fold induction of microarray data.
