# Supplementary material for: Xanthomonas oryzae pv oryzae triggers immediate transcriptomic modulations in rice
Source: BMC Genomics. 2012 Jan 31;13:49. doi: 10.1186/1471-2164-13-49 (PMC3298507; doi:10.1186/1471-2164-13-49)
Supplement: Additional file 5 — Interaction network of up-regulated transcripts. A powerpoint file containing interaction map generated by Pathway Studio (version 7.1). The transcripts up-regulated in present study are highlighted in yellow. [file 1471-2164-13-49-S5.PPT]

## Slide 1
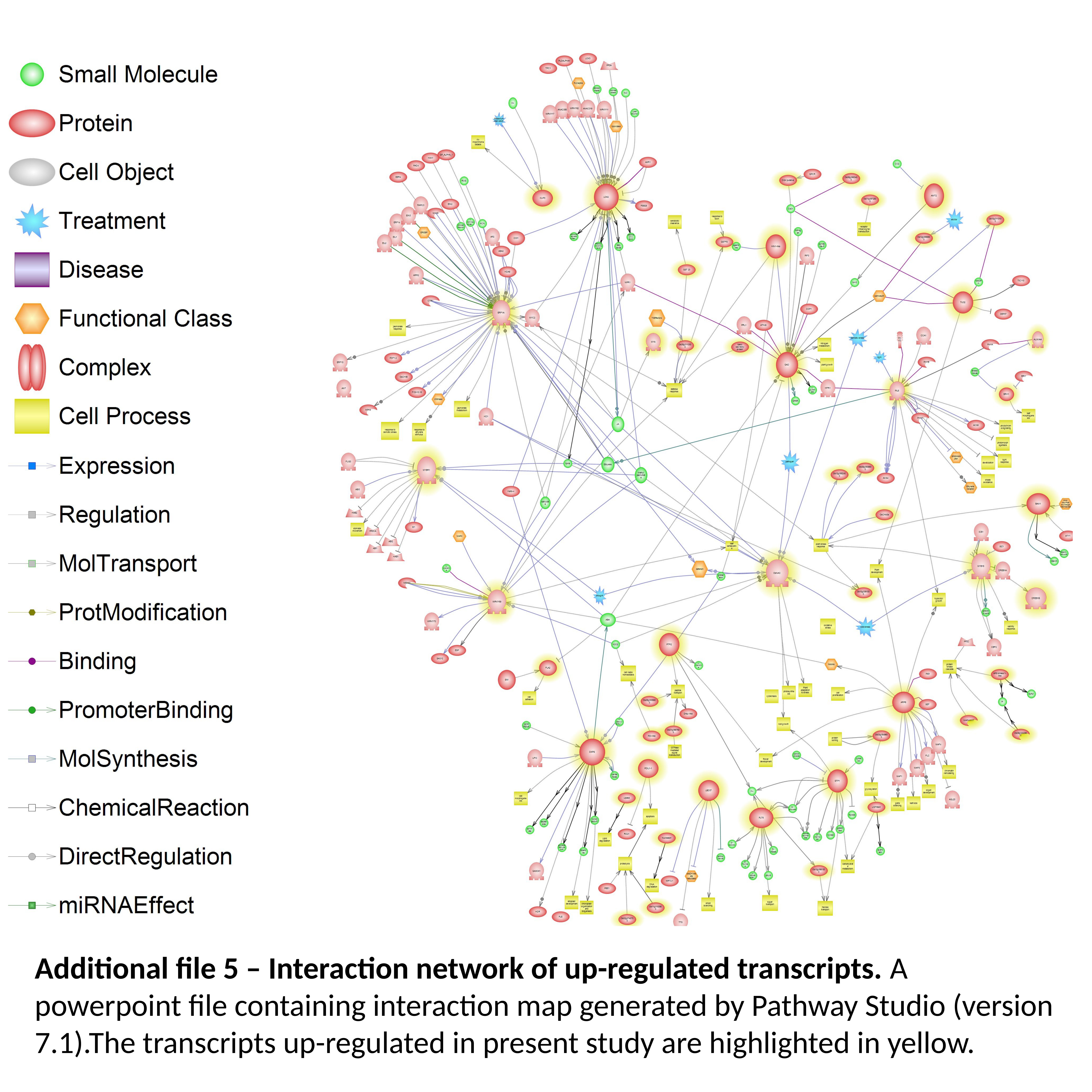

Additional file 5 – Interaction network of up-regulated transcripts. A powerpoint file containing interaction map generated by Pathway Studio (version 7.1).The transcripts up-regulated in present study are highlighted in yellow.
