# Supplementary material for: Xanthomonas oryzae pv oryzae triggers immediate transcriptomic modulations in rice
Source: BMC Genomics. 2012 Jan 31;13:49. doi: 10.1186/1471-2164-13-49 (PMC3298507; doi:10.1186/1471-2164-13-49)
Supplement: Additional file 7 — K means clusters comparing the expressional level of transcripts. A powerpoint file containing K means clusters comparing the expression level of transcripts having beyond +/-2 fold change and p < = 0.05 at 0,1,6 and 120 hours after inoculation. The green and orange represent down-regulation and up-regulation respectively. The intensity of color signifies the degree of fold change. [file 1471-2164-13-49-S7.PPT]

## Slide 1
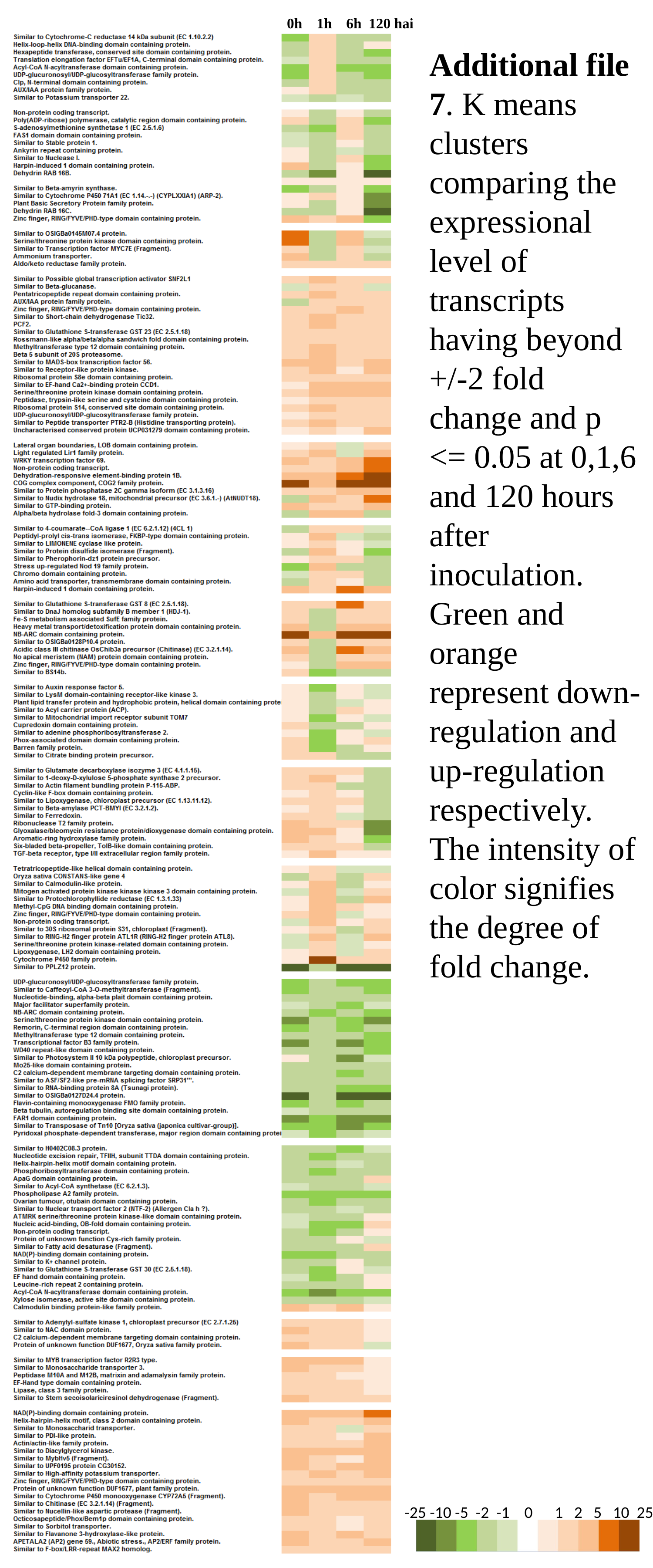

0h 1h 6h 120 hai
Additional file 7. K means clusters comparing the expressional level of transcripts having beyond +/-2 fold change and p <= 0.05 at 0,1,6 and 120 hours after inoculation. Green and orange represent down-regulation and up-regulation respectively. The intensity of color signifies the degree of fold change.
-25 -10 -5 -2 -1 0 1 2 5 10 25
