# Supplementary material for: Xanthomonas oryzae pv oryzae triggers immediate transcriptomic modulations in rice
Source: BMC Genomics. 2012 Jan 31;13:49. doi: 10.1186/1471-2164-13-49 (PMC3298507; doi:10.1186/1471-2164-13-49)
Supplement: Additional file 9 — The representative data of microarray hybridization and qRT-PCR. A powerpoint file containing A. Part of image of microarray hybridization. B. Scatter plot of log intensities across an array. C. Real-time PCR curve. [file 1471-2164-13-49-S9.PPT]

## Slide 1
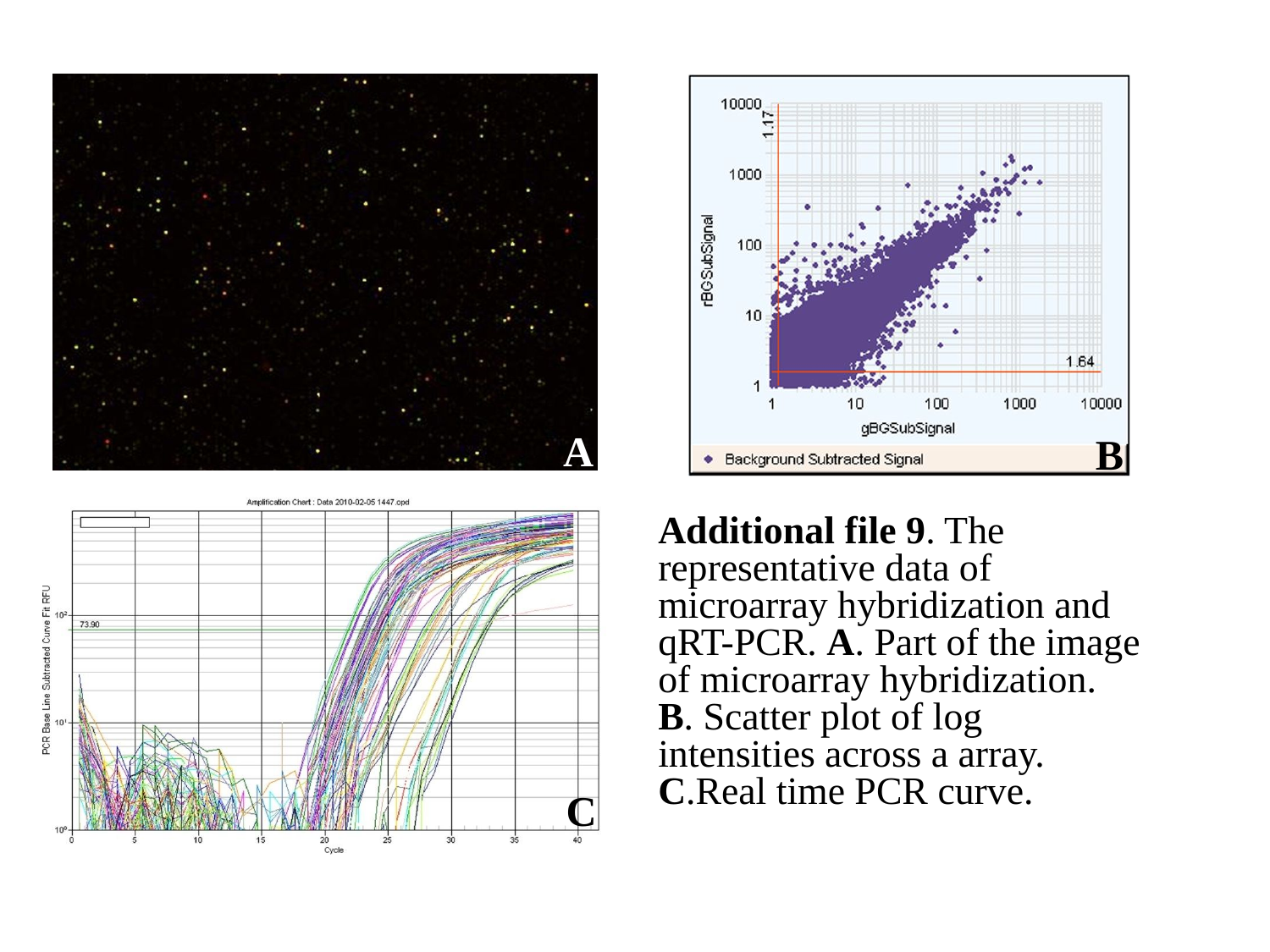

A
B
# Additional file 9. The representative data of microarray hybridization and qRT-PCR. A. Part of the image of microarray hybridization. B. Scatter plot of log intensities across a array. C.Real time PCR curve.
C
